# Nonlinear image registration with bidirectional metric and reciprocal regularization

**DOI:** 10.1371/journal.pone.0172432

**Published:** 2017-02-23

**Authors:** Shihui Ying, Dan Li, Bin Xiao, Yaxin Peng, Shaoyi Du, Meifeng Xu

**Affiliations:** 1 Department of Mathematics, Shanghai University, Shanghai 200444, China; 2 Med-X Research Institute, School of Biomedical Engineering, Shanghai Jiao Tong University, Shanghai 200240, China; 3 Institute of Artificial Intelligence and Robotics, Xi’an Jiaotong University, Xi’an 710049, China; 4 The Second Affiliated Hospital of Xi’an Jiaotong University, Xi’an 710004, China; Chinese Academy of Sciences, CHINA

## Abstract

Nonlinear registration is an important technique to align two different images and widely applied in medical image analysis. In this paper, we develop a novel nonlinear registration framework based on the diffeomorphic demons, where a reciprocal regularizer is introduced to assume that the deformation between two images is an exact diffeomorphism. In detail, first, we adopt a bidirectional metric to improve the symmetry of the energy functional, whose variables are two reciprocal deformations. Secondly, we slack these two deformations into two independent variables and introduce a reciprocal regularizer to assure the deformations being the exact diffeomorphism. Then, we utilize an alternating iterative strategy to decouple the model into two minimizing subproblems, where a new closed form for the approximate velocity of deformation is calculated. Finally, we compare our proposed algorithm on two data sets of real brain MR images with two relative and conventional methods. The results validate that our proposed method improves accuracy and robustness of registration, as well as the gained bidirectional deformations are actually reciprocal.

## 1 Introduction

Magnetic Resonance Imaging (MRI) technique plays more and more important roles in the study of brain structure and its function because it offers amount of reliable information by a non-invasive approach [1]. Due to the large variances between the brain images from different subjects, we cannot compare or analyze different MR images directly and should first align them to a common reference. Therefore, normalizing the images is a precondition for the clinical research [2–5].

In recent decades, a variety of effective registration methods are developed for constructing the common reference and calculating the deformations between different images. Especially, as a kind of fundamental methods, pairwise registration plays important roles in variants of registration problems. According to the differences between representations of model and algorithm, these methods are divided into two classes. One is the parametrization methods, in which the deformation between two images is approximated in some finite dimensional spaces. For example, Shen and Davatzikos proposed the HAMMER algorithm based on the process of feature detection and hierarchical deformation mechanism [6] and the further improved version, named TPS-HAMMER [7] by introducing thin-plate spline and softassign techniques [8]. Sorzano and his coauthors used the B-Spline method to approximate the nonlinear deformation [9] while Rohde and his coauthors adopted the radially symmetric basis [10]. The others are the variational models and methods, where the deformation is regarded as a certain functional and the optimal deformation is represented by variational calculus. For example, a series of nonlinear registration methods are developed by using an diffeomorphism to represent the deformation [11–23] based on the fundamental work by Dupuis, Grenander, Miller and Trouvé [24, 25]. By the diffeomorphic representation, a uniform framework for pairwise image registration is constructed and later researches mainly focused on improving the model and how to solve it efficiently. It should be pointed out that Beg and his coauthors proposed the large deformation diffeomorphic metric mapping (LDDMM) method by the variation method and geodesic shooting strategy [14], which gives a fine mathematical framework, while Cao [15], Qiu [19] and Sommer [20] developed this method and applied them in different issues, respectively. At the same time, Trouvé, Holm and Younes considered the nonlinear registration on the diffeomorphism group directly and formed a metamorphosis theory [17, 21], where the existence of solution of Euler equations was proved. For simplifying the registration model, Ashburner decomposed the large deformation into several small deformations and translated the image registration into a local optimisation problem which is solved by a Levenberg-Marquardt strategy [11]. On the other hand, some effective numerical methods for these registration models were developed. Vercauteren and his coauthors combined the Demons algorithm with the diffeomorphic framework and proposed Diffeomorphic Demons algorithm [22], while they added the high-order information to their model by using the high-order BCH formulae [23]. A comparison of LDDMM and Diffeomorphic Demons can be found in [16]. As a conclusion, Diffeomorphic Demons algorithm is much faster than LDDMM algorithm, while it incurs moderate loss of accuarcy. Later, Ashburner and Friston used the Gauss-Newton iteration to solve the diffeomorphic registration model [12]. Klein and his coauthors compared more recent 14 registration methods in [26]. For a comprehensive review of image registration and especially diffeomorphism methods, we refer to [27] and [28, 29], respectively.

Although these algorithms can deal with the nonlinear image registration, there are still two issues. One is the invertibility of the deformation field between two images in practice and the other is the accuracy of registration. Due to various approximations in the model and calculation, the deformation is always not an exact diffeomorphism. That is, the composition of the deformation and its inverse deformation is not an exact identity [30]. To deal with these problems, Ye and Chen introduced a constraint to deformation and designed an algorithm based on a numerical PDE method [30]. Later, Lorenzi and his coauthors proposed a fast symmetric method (LCC-Demons) based on Diffeomorphic Demons by using a symmetric local correlation coefficient (LCC) metric and one order approximations of the deformation and its inversion [18]. It should be pointed out that, neither Ye and Chen’s method nor LCC-Demons settled down the above two issues thoroughly. Therefore, in this paper, we will further address these two issues by using the advantages of these two methods. First, we apply a bi-direction sum of squire distance (SSD) metric instead of the conventional SSD metric, which introduces the deformation and its inverse synchronously. Then, we introduce an exact diffeomorphic constraint and penalize it to the objective function. Finally, inspired by the solution of Diffeomorphic Demons, we calculate the closed form of velocity at each iteration and design a novel nonlinear registration algorithm, in which a multi-scale strategy and a step-by-step warping process [31] are used.

The remainder of this paper is organized as follows. In Section 2, we first recall some relative materials for diffeomorphic registration methods, and then we extend the model of Diffeomorphic Demons by using a bi-direction metric and introducing the reversible constraint. Then, in Section 3, we propose a solution of such new model and form a novel registration algorithm. To validate the efficiency of proposed algorithm, we compare it with the conventional Diffeomorphic Demons and symmetric Diffeomorphic Demons methods in Section 4. Finally, the whole paper is concluded in Section 5.

## 2 Symmetric demons model with a reciprocal regularization

In this section, we first recall the diffeomorphic model for the nonlinear image registration and the Diffeomorphic Demons method. Then, we introduce a symmetric metric, which is widely used in computer vision, and a group of symmetrically reversible constraint to the model.

Denote the region of the image by $\Omega \subset R^{n}$ (where *n* is always 2 or 3), and then an image is always represented by a BV function on this region. For a fixed image *F* and a moving image *M*, the aim of registration is to find the best deformation filed *s* from *M* to *F*, and the general model of nonlinear registration is described as follows.

$$s^{*}=\underset{s\in \text{Diff}(\Omega )}{\arg\min}\text{Dist}\left(F,s\cdot M\right)+\lambda \text{Reg}\left(s\right),$$ (1)

where Diff(Ω) = {*s*|*s*, *s*^−1^ ∈ *C*^∞^(Ω, Ω)} is the set of all invertible and smooth deformations from the region Ω to itself, Dist(⋅, ⋅) is the distance to measure the similarity between two images, Reg(*s*) is the regular term of the deformation *s*, *s* ⋅ *M* means the deformation *s* acting on the image *M* and is defined by (*s* ⋅ *M*)(*x*) = *M*(*s*(*x*)), ∀*x* ∈ Ω, and *λ* is a balancing parameter. It should be pointed out that there are several selections of the similarity metric and the regularity term. In this paper, we adopt an *L*^2^ metric (SSD between two images) as the similarity metric, which is defined by

$$\text{Dist}\left(F,s\cdot M\right)=\int_{\Omega }{\left|F\left(x\right)-M\left(s\left(x\right)\right)\right|}^{2}dx,$$ (2)

and the regularity term Reg(*s*) is selected as the Laplacian regularizer and defined by

$$\text{Reg}\left(s\right)={∥\nabla s∥}^{2}.$$ (3)

To improve the precision of registration and consider the inverse deformation field, Vercauteren et al. introduced a bi-direction metric instead of the conventional SSD in [23]. That is, the similarity metric in Eq (1) is revised to

$$\text{Dist}\left(F,s\cdot M\right)+\text{Dist}\left(M,s^{-1}\cdot F\right),$$ (4)

where *s*^−1^ is the deformation from *F* to *M* and should be the inverse of *s*. In [23], to make the algorithm faster, the authors used the linearization of *s* and its opposite to approximate the deformations *s* and *s*^−1^, and hence the composition of deformations from *F* to *M* and from *M* to *F* is not exact identity. Therefore, in order to make the deformation being a exact diffeomorphism and keep the advantages of Diffeomorphic Demons, we slack the symmetric registration model by replacing the *s*^−1^ by an independent deformation *t* and add a Reciprocal constraint *s* ∘ *t* = *t* ∘ *s* = Id to the model. Then, the registration model is translated to the following minimization problem.

$$\min_{s,t}E\left(s,t\right)≔\text{Dist}\left(F,s\cdot M\right)+\text{Dist}\left(M,t\cdot F\right)+\lambda \left[\text{Reg}\left(s\right)+\text{Reg}\left(t\right)\right]$$ (5)

s.t.s∘t=Id,t∘s=Id,∀s,t∈Diff(Ω) (6)

where *λ* is a positive parameter, ∘ is the composite operator of two deformations and Id is the identity deformation.

Combining the similarity metric in Eq (2) and regularity term in Eq (3), the final symmetric registration model can be represented by

$$\;\min_{s,t}E\left(s,t\right)≔\int_{\Omega }\left[{\left|F\left(x\right)-M\left(s\left(x\right)\right)\right|}^{2}+{\left|M\left(x\right)-F\left(t\left(x\right)\right)\right|}^{2}\right]\;dx+{\lambda (∥\nabla s∥}^{2}+{∥\nabla t∥}^{2})$$ (7)

s.t.t∘s=Id,s∘t=Id,∀s,t∈Diff(Ω) (8)

## 3 Algorithm

To design the algorithm, we first penalize the constraint to the objective function. Then, problems (7)–(8) is translated to following unconstrained optimization problem.

$$\;\min_{s,t,\mu }\int_{\Omega }{\left|F\left(x\right)-M\left(s\left(x\right)\right)\right|}^{2}{+|M\left(x\right)-F\left(t\left(x\right)\right)|}^{2}dx+{\lambda (∥\nabla s∥}^{2}+{∥\nabla t∥}^{2}{)+\mu (∥\text{Id}-t\circ s∥}^{2}{+∥\text{Id}-s\circ t∥}^{2})$$ (9)

where the positive parameter *μ* is the Lagrange multiplier. It should be pointed out that we only use one multiplier here because of the dependence of two constraints. That is, one constraint includes the other one. Therefore, in practice, we only use one constraint.

It is clear that there are two independent variables except for *μ* in the objective function in Eq (9), therefore the simplest method to solve such minimizing problem is the alternating iterative method. That is, the minimizing problem (9) can be solved by alternatively iterating the following two subproblems.

**(S1)** Fixing current deformation *s*^*k*^, we solve the *k*^*th*^ deformation *t*^*k*^ from *F* to *M* by
  $$\;\min_{t\in \text{Diff}(\Omega )}E_{1}^{k}\left(t\right)≔\int_{\Omega }{\left|M\left(x\right)-F\left(t\left(x\right)\right)\right|}^{2}dx+{\lambda ∥\nabla t∥}^{2}+\mu {∥\text{Id}-s^{k}\circ t∥}^{2}$$ (10)
**(S2)** Given the deformation *t*^*k*^, we calculate the (*k* + 1)^*th*^ deformation *s*^*k*+1^ from *M* to *F* by
  $$\;\min_{s\in \text{Diff}(\Omega )}E_{2}^{k}\left(s\right)≔\int_{\Omega }{\left|F\left(x\right)-M\left(s\left(x\right)\right)\right|}^{2}dx+{\lambda ∥\nabla s∥}^{2}+\mu {∥\text{Id}-t^{k}\circ s∥}^{2}$$ (11)

It is worth mentioning that the first two terms of Eqs (10) and (11) are similar to the objective function in Diffeomorphic Demons method. The only difference is the third term, which assures that the composition of forward and backward deformation is identity. Therefore, we can inherit the advantages of Diffeomorphic Demons to design an algorithm.

We consider the subproblem Eq (11) first and subproblem Eq (10) can be solved in the same way. It should be pointed out that it is difficult to solve the subproblem Eq (11) directly by minimizing the the similarity and regularity term simultaneously. Therefore, as Diffeomorphic Demons, we rewrite the model by introducing an independent intermediate variable *c*, and Eq (11) is equivalent to the following minimizing problem,

$$\begin{array}{ccc} & & \;\min_{s\in \text{Diff}(\Omega )}E_{2}^{k}\left(s\right)≔\int_{\Omega }{\left|F\left(x\right)-M\left(c\left(x\right)\right)\right|}^{2}dx+{\lambda ∥\nabla s∥}^{2}+\mu {∥\text{Id}-t^{k}\circ c∥}^{2}, \end{array}$$

s.t.c=s

Still by Lagrange multiplier method, the above minimizing problem can be translated to the following unconstrained optimization problem

$$\begin{array}{ccc} & & \;\min_{s,c\in \text{Diff}(\Omega )}E_{2}^{k}\left(s,c\right)≔\int_{\Omega }{\left|F\left(x\right)-M\left(c\left(x\right)\right)\right|}^{2}dx+{\lambda ∥\nabla s∥}^{2}+\mu ∥\text{Id}-t^{k}{\circ c∥}^{2}+\sigma {∥c-s∥}^{2}, \end{array}$$

where *σ* is a positive parameter used to describe the weight of similarity between *c* and *s*. Then, to deal with such optimization problem with two independent variables, we can also adopt an efficient two-step process alternatively as follows.

**(R1)** Fixing current fixed deformation *s*^*k*,*l*^, we solve the intermediate variable *c*^*k*,*l*^ by
  $$\;\min_{c\in \text{Diff}(\Omega )}H_{2,1}^{k}\left(c\right)≔\int_{\Omega }{\left|F\left(x\right)-M\left(c\left(x\right)\right)\right|}^{2}dx+\mu ∥\text{Id}-t^{k}{\circ c∥}^{2}+\sigma {∥c-s^{k,l}∥}^{2},$$ (12)
**(R2)** Given the the intermediate variable *c*^*k*,*l*^, we calculate the *s*^*k*,*l*+1^ by
  $$\min_{s\in \text{Diff}(\Omega )}H_{2,2}^{k}\left(s\right)≔{\lambda ∥\nabla s∥}^{2}+\sigma {∥c^{k,l}-s∥}^{2}.$$ (13)

Here, we use *k* and *l* to represent the indices of the outer iteration for Eqs (10) and (11), and inner iteration for Eqs (12) and (13), respectively. It is worth mentioning that we can use a Gaussian Kernel to smooth the deformation *c*^*k*,*l*^ to solve the problem (13) because the meaning of Eq (13) is to find a deformation which is similar to *c*^*k*,*l*^ as well as smooth enough [32]. Then, below we only consider the problem (12). The direct way is to use the gradient descent method. Moreover, because the deformations are in the diffeomorphism group, we use an intrinsic iteration as follows.

$$c^{k,l}=s^{k,l}\circ \exp\left(u^{k,l}\right)$$ (14)

where *u*^*k*,*l*^ is a velocity of the deformation, which is located in the tangent space of diffeomorphism group at *s*^*k*,*l*^, and exp is the exponential map. Therefore, the rest issue is how to solve the *u*^*k*,*l*^. By this representation, the optical flow procedure solves for the below energy function.

$$\;\min_{u\in X(\text{Diff}(\Omega ))}H_{2,1}^{k}\left(u\right)=\int_{\Omega }|F\left(x\right)-M\left(s^{k,l}\circ \exp\left(u\right)\left(x\right)\right){|}^{2}dx+\sigma ∥s^{k,l}\circ \exp\left(u\right)-s^{k,l}{∥}^{2}+\mu {∥\text{Id}-t^{k}\circ s^{k,l}\circ \exp\left(u\right)∥}^{2}$$ (15)

where X(Diff(Ω)) is the set of all vector fields on the Diff(Ω).

To make two parts of the functional unified with respect to the order of magnitudes in the objective function in Eq (15), we use the weak form in the constraint part. That is, we consider the identity by acting on an image, which also makes programming easier. Then, Eq (15) is translated to

$$\;\min_{u\in X(\text{Diff}(\Omega ))}L_{2}^{k}\left(u\right)≔\int_{\Omega }|F\left(x\right)-M\left(s^{k,l}\circ \exp\left(u\right)\left(x\right)\right){|}^{2}dx+\mu \int_{\Omega }|F\left(x\right)-F\left(t^{k}\circ s^{k,l}\circ \exp\left(u\right)\left(x\right)\right){|}^{2}+\sigma {∥s^{k,l}\circ \exp\left(u\right)-s^{k,l}∥}^{2}$$ (16)

To solve Eq (16) easier, similar to the Diffeomorphic Demons, we use the first order approximation to the exponential map and substitute it into the objective function (16). Therefore, we use the first order Taylor formula of any differentiable functional *f* on Diff(Ω) at a neighborhood of the deformation *s* as follows.

$$f\left(s\circ \exp\left(u\right)\right)=f\left(s\right)+J_{s}^{f}\cdot u+{O(∥u∥}^{2})$$ (17)

where

$${\left[J_{s}^{f}\right]}_{i}=\frac{\partial }{\partial u_{i}}f\left[s\circ \exp\left(u\right)\right]{|}_{u=0}$$

is the gradient of the functional *f* at *s*.

By this approximation, the objective function in Eq (16) is approximated by

$$\;L_{2}^{k}\left(u\right)\approx \int_{\Omega }{\left|\left[F\left(x\right)-M\left(s^{k,l}\left(x\right)\right)\right]-J_{s^{k,l}}^{M}\left(x\right)\cdot u\left(x\right))\right|}^{2}dx+\mu \int_{\Omega }{\left|\left[F\left(x\right)-F\left(t^{k}\circ s^{k,l}\left(x\right)\right)\right]-J_{t^{k}\circ s^{k,l}}^{F}\left(x\right)\cdot u\left(x\right))\right|}^{2}dx+\sigma {∥u∥}^{2}$$ (18)

It is seen that this approximated objective function is *L*^2^ for *u*. Therefore, to make the equation short, we rewrite it as follows,

$$L_{2}^{k}\left(u\right)\approx \int_{\Omega }{∥\left(\begin{array}{c} FM\\ 0\\ FF \end{array}\right)-\left(\begin{array}{c} J_{s^{k,l}}^{M}\\ \sqrt{\sigma /|\Omega |}\\ \sqrt{\mu }\cdot J_{t^{k}\circ s^{k,l}}^{F} \end{array}\right)\cdot u∥}^{2}dx$$ (19)

where

$$FM\left(x\right)=F\left(x\right)-M(s^{k,l}\left(x\right)),$$

$$FF\left(x\right)=F\left(x\right)-F(t^{k+1}\circ s^{k,l}\left(x\right)),$$

and |Ω| is the volume of the image region Ω.

Moreover, minimizing Eq (19) is equivalent to solving the following linear system for *u*.

$$\left[\begin{array}{c} J_{s^{k,l}}^{M}\\ \sqrt{\sigma /|\Omega |}\\ \sqrt{\mu }\cdot J_{t^{k}\circ s^{k,l}}^{F} \end{array}\right]\;u\left(x\right)=\left[\begin{array}{c} FM(x)\\ 0\\ FF(x) \end{array}\right]$$ (20)

According to the generalized inverse and the Sherman–Morrison formula, we obtain a closed form of the velocity vector at the point *x* ∈ Ω as follows,

$$u^{k,l}\left(x\right)=\frac{\left[FM\left(x\right)\right]J_{s^{k,l}}^{M}\left(x\right)+\sqrt{\mu }\left[FF\left(x\right)\right]J_{t^{k}\circ s^{k,l}}^{F}\left(x\right)}{∥J_{s^{k,l}}^{M}{\left(x\right)∥}^{2}+\sigma /|\Omega |+\mu ∥J_{t^{k}\circ s^{k,l}}^{F}{\left(x\right)∥}^{2}}.$$ (21)

Similarly, we can solve subproblem Eq (10) by only changing the corresponding variables in iterations as follows.

$$t^{k,l+1}=K⋆\left[t^{k,l}\circ \exp\left(v^{k,l}\right)\right]$$ (22)

where *K*⋆ is a Gaussian kernel *K* acting on the deformation, and

$$v^{k,l}\left(x\right)=\frac{\left[MF\left(x\right)\right]J_{t^{k,l}}^{F}\left(x\right)+\sqrt{\mu }\left[MM\left(x\right)\right]J_{s^{k}\circ t^{k,l}}^{M}\left(x\right)}{∥J_{t^{k,l}}^{F}{\left(x\right)∥}^{2}+\sigma /|\Omega |+\mu ∥J_{s^{k}\circ t^{k,l}}^{M}{\left(x\right)∥}^{2}}$$ (23)

where

$$MF\left(x\right)=M\left(x\right)-F(t^{k,l}\left(x\right)),$$

$$MM\left(x\right)=M\left(x\right)-M(s^{k}\circ t^{k,l}\left(x\right)),$$

and $J_{t^{k,l}}^{F}=\nabla \left(t^{k,l}\cdot F\right)$ and $J_{s^{k}\circ t^{k,l}}^{M}=\nabla \left(s^{k}\circ t^{k,l}\cdot M\right)$ are the corresponding Jacobi of two terms in Eq (10).

Therefore, we summarize the algorithm as follows.

Algorithm 1. Constrained Symmetric Algorithm

Step 0. Initialization.

   Given the precision *ϵ*_1_, *ϵ*_2_ > 0 and initial deformation fields *s*^1^ and *t*^1^.

Step 1. Solving the minimization problem (10) by setting *t*^*k*,0^ = *t*^*k*^,

 1.1 Calculating *v*^*k*,*l*^ by Eq (23),

 1.2 Smoothing the updated field by a Gaussian Kernel: *v*^*k*,*l*^ ← *K*⋆*v*^*k*,*l*^,

 1.3 Set *t*^*k*,*l*+1^ = *t*^*k*,*l*^ ∘ exp(*v*^*k*,*l*^),

 1.4 If ‖*v*^*k*,*l*^‖ > *ϵ*_1_ then set *l* ⇐ *l* + 1 and goto Step 1.1. Otherwise let *t*^*k*+1^ = *t*^*k*,*l*^ and goto Step 2;

Step 2. Solving the minimization problem (11) by setting *s*^*k*,0^ = *s*^*k*^,

 2.1 Calculating *u*^*k*,*l*^ by Eq (21),

 2.2 Smoothing the updated field by a Gaussian Kernel: *u*^*k*,*l*^ ← *K*⋆*u*^*k*,*l*^,

 2.3 Set *s*^*k*,*l*+1^ = *s*^*k*,*l*^ ∘ exp(*u*^*k*,*l*^),

 2.4 If ‖*u*^*k*,*l*^‖ > *ϵ*_1_ then set *l* ⇐ *l* + 1 and goto Step 2.1. Otherwise let *s*^*k*+1^ = *s*^*k*,*l*^ and goto Step 3;

Step 3. If *E*(*s*^*k*+1^, *t*^*k*+1^) > *ϵ*_2_, then *k* ⇐ *k* + 1 and goto Step 1. Otherwise output the optimal deformations s* = *s*^*k*+1^ and t* = *t*^*k*+1^.

It is remarkable that the deformation is updated by combining the last deformation and the exponential map of a velocity field. In fact, the exponential map of a vector field is difficult to be calculated, especially when the velocity field is large. Here, we use a kind of Arsigny’s approximate method. For the other approximate methods for the exponential map (or geodesic), we refer to [14, 33]. First, we evaluate the velocity field *u*. If it is too large, we choose a positive integer *N* such that *u*/2^*N*^ is significantly small. Then, exp(*u*/2^*N*^) ≈ Id + *u*/2^*N*^, and $\text{exp}\left(u\right)\approx \underset{2^{N}}{\underset{︸}{\text{exp}\left(u/2^{N}\right)\circ \cdots \circ \text{exp}\left(u/2^{N}\right)}}$.

On the other hand, to accelerate the algorithm and avoid the local minima as possible, a multiscale strategy is used. We estimate the deformation between two images from the low to high resolution, and the deformation field in the low resolution is used as the initial deformation for the high resolution.

## 4 Experimental results

In this section, we evaluate the performance of our proposed registration algorithm on two real datasets of LONI LPBA40 MR images and IXI MR images. For the sake of comparison, we also use the Diffeomorphic Demons [22] and the symmetric Diffeomorphic Demons [18]. All programs are implemented in visual studio 2008, ITK2.4.0 and run by PC with Intel Core i7 2.50GHz CPU and 4G RAM.

### 4.1 LONI LPBA40 data

**Fig 1:**
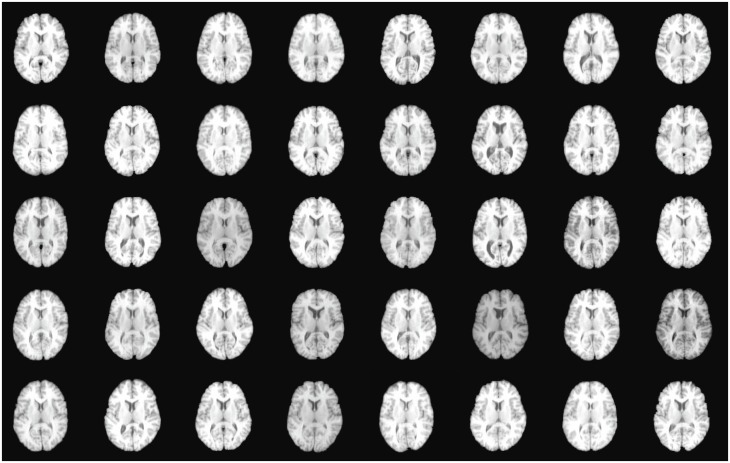
40 LONI MR images.

In order to test our registration algorithm, we compare with the Diffeomorphic Demons and Symmetric-log-Demons algorithms on a public LONI dataset, which is widely used for testing registration algorithms. There are totally 40 MR images shown in Fig 1, and their labels used to construct the LONI Probabilistic Brain Atlas (LPBA40) at the Laboratory of Neuro Imaging (LONI) at UCLA are available online (http://www.loni.usc.edu/atlases/Atlas_Detail.php?atlas_id=12). In each subject, 56 structures (ROIs) were manually labeled and brain masks were constructed from the manual labels and projected back to the labeling space to produce brain only MRI volumes.

**Fig 2:**
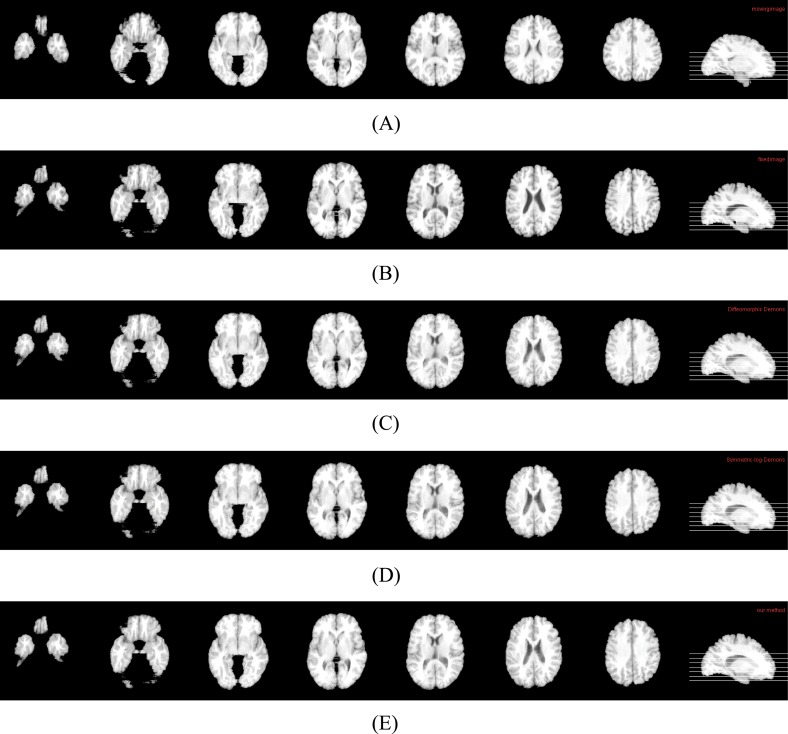
Cross-sectional views of data and registration results by three methods. (A) Cross-sectional views of moving image. (B) Cross-sectional views of fixed image. (C) Cross-sectional views of the result of Diffeomorphic Demons. (D) Cross-sectional Views of the result of Symmetric-log-Demons. (E) Cross-sectional Views of the result of our method.

**Fig 3:**
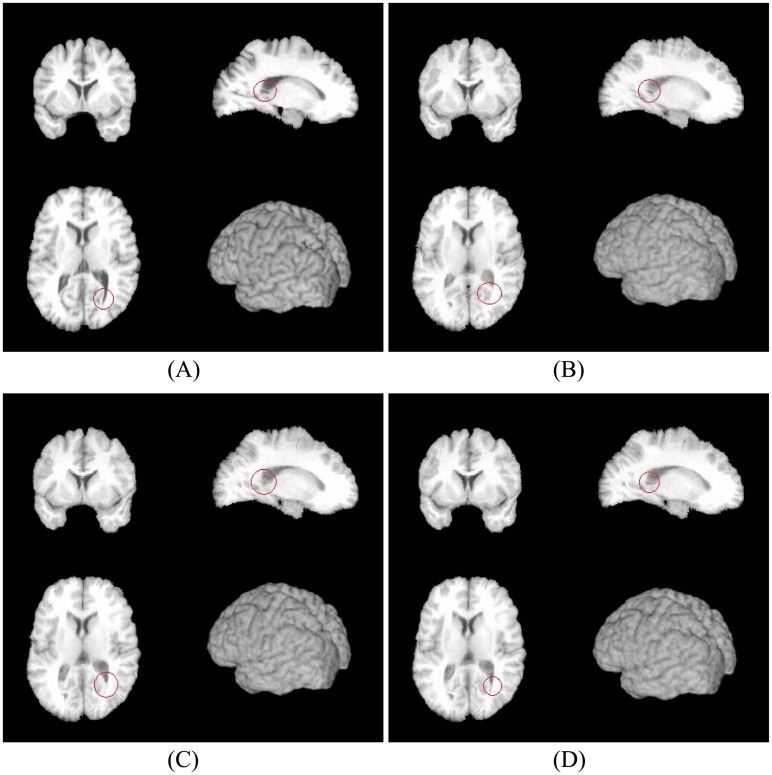
Fixed image and the registration results by three methods. (A) Fixed image. (B) Registration result by Diffeomorphic Demons. (C) Registration result by Symmetric-log-Demons. (D) Registration result by our method.

**Fig 4:**
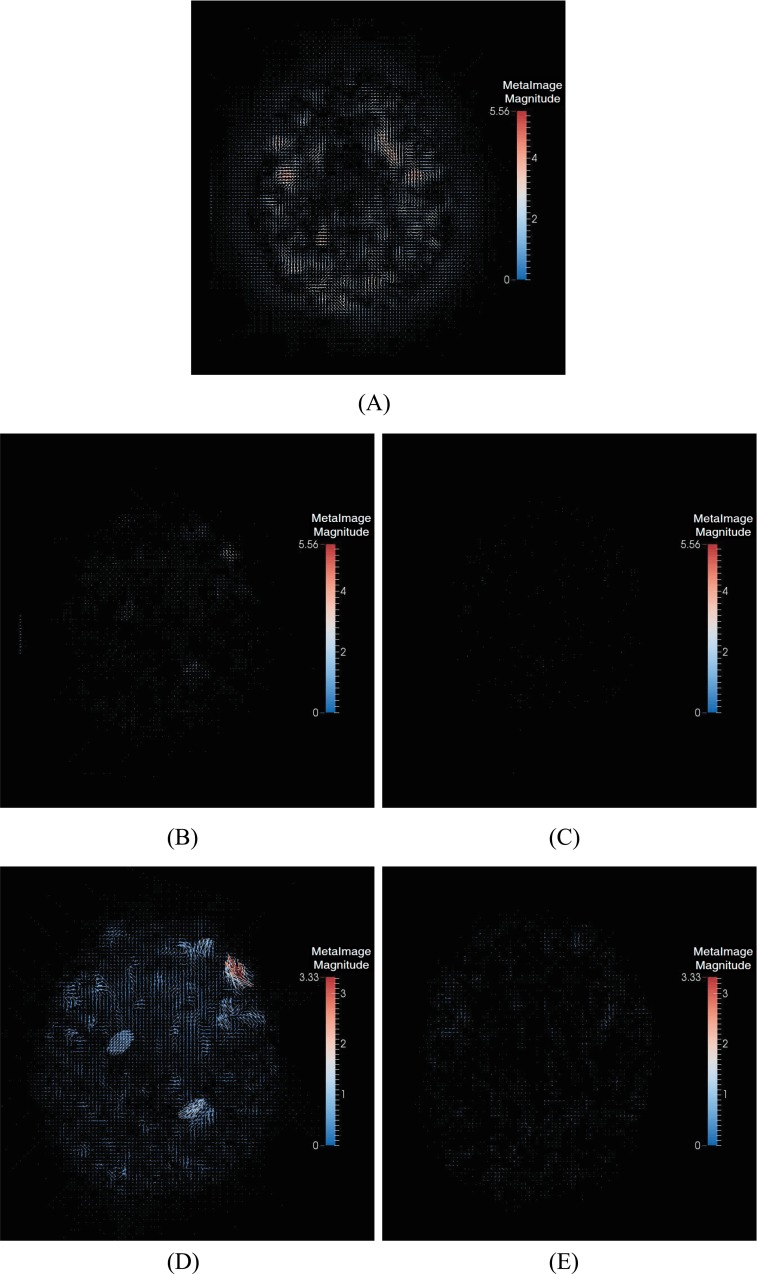
The combined deformations by three methods. (A) Diffeomorphic Demons. (B) Symmetric-log-Demons. (C) our method. (D) Symmetric-log-Demons in 10 times magnification. (E) Our method in 10 times magnification.

**Fig 5:**
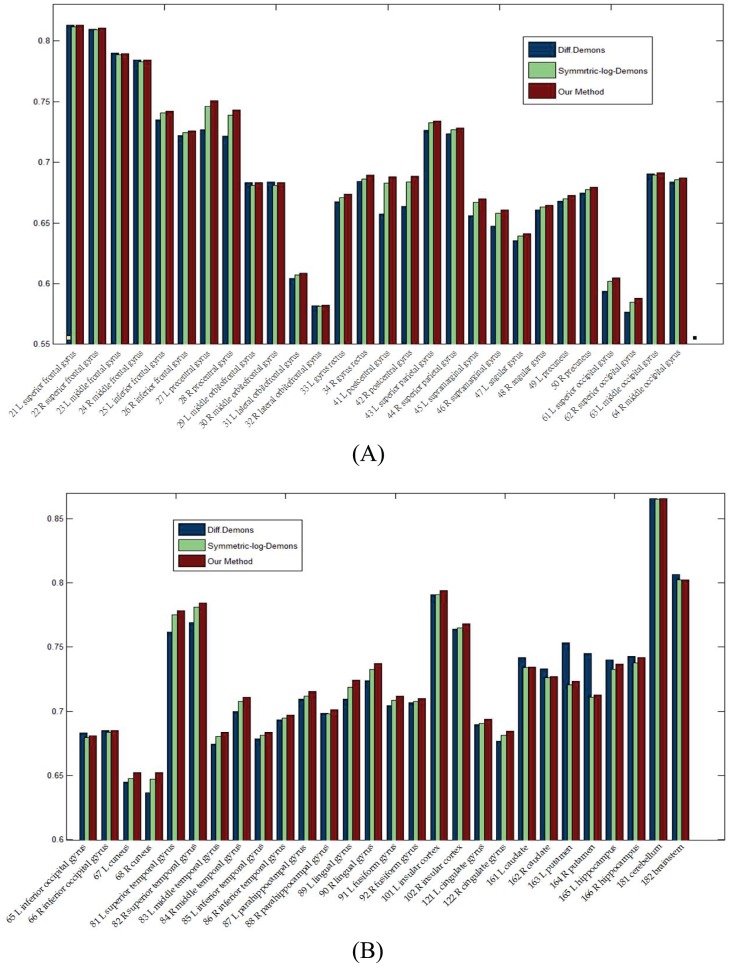
Dice ratios of 56 ROIs by three methods. (A) Dice ratios from 1st to 28th ROIs. (B) Dice ratios from 29th to 56th ROIs.

By using two conventional nonlinear registration methods and our method, one group of results are shown in Figs 2, 3 and 4, and the quantitative results are shown in Table 1 and Fig 5.

**Table 1:** Dice ratios of three ROIs by three methods.

	whole brain (average)
Diff. Demons	74.28 ± 2.0991
Symmetric-Demons	74.63 ± **1.9787**
Our method	**74.84** ± 2.0080

In Fig 2, (A) and (B) are the cross-sectional images of the moving image and the fixed image, respectively. (C), (D) and (E) are the registration results from the moving image to the fixed image by three algorithms. It is seen that all methods do the registration well. That is, (C), (D) and (E) are all similar to (B). It should be pointed out that there are still have some differences, which are shown in Fig 3. It is easy to find that our registration result (D) still has a slight improvement and is closer to the fixed image (A).

On the other hand, we show the combinations of the straight and backward deformation fields in Fig 4. From Fig 4(A), 4(B) and 4(C), the maximal magnitude is 5.56 mm. At this scale, (B) and (C) are almost black. That is, the combinations of the straight and backward deformation fields are closer to identities. Therefore, it is seen that both Symmetric-Demons and our proposed method are more accurate than the conventional Diffeomorphic Demons. But when we enlarge (b) and (C) with scale 10, the results are shown in Fig 4(D) and 4(F). From the Fig 4(D), the maximal magnitude of Symmetric-Demons is 3.33 mm, and the combined deformation field of our method is still close to an identity. That is, the deformation of our proposed method is closer to the diffeomorphism than the Symmetric-Demons, and therefore, our proposed method is more accurate.

Finally, to quantitatively evaluate the registration accuracy, we calculate the Dice ratios of 56 ROIs by three methods shown in Fig 5 and the total average of whole brain is list in Table 1. It is clear that our proposed method has a slight improvement.

### 4.2 IXI data

**Fig 6:**
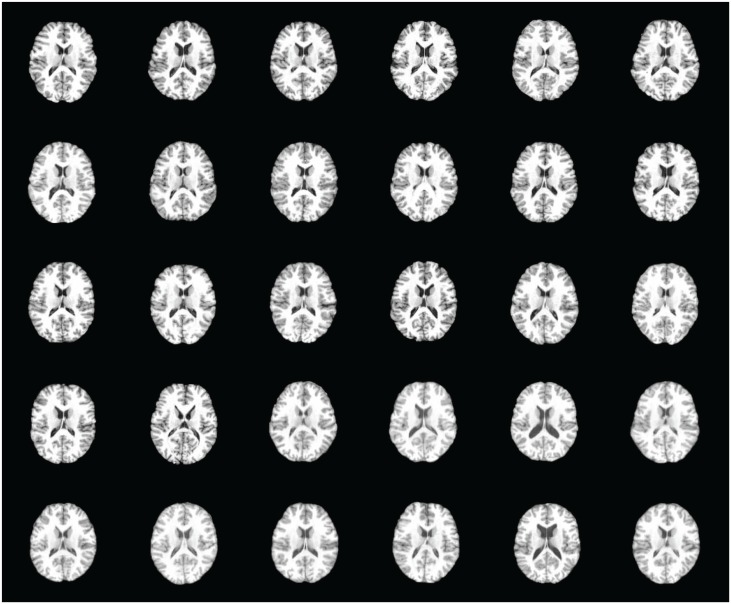
30 IXI MR images.

In this part, we conduct the same experiment on the data of IXI MR images, which can be downloaded at http://brain-development.org/ixi-dataset/. Here, we use 30 MR images in the following experiment. Each image has 83 manually delineated ROIs and its size of is 256 × 256 × 198 and the voxel spacing is 1 × 1 × 1mm^3^. Slices of images are shown in Fig 6.

**Fig 7:**
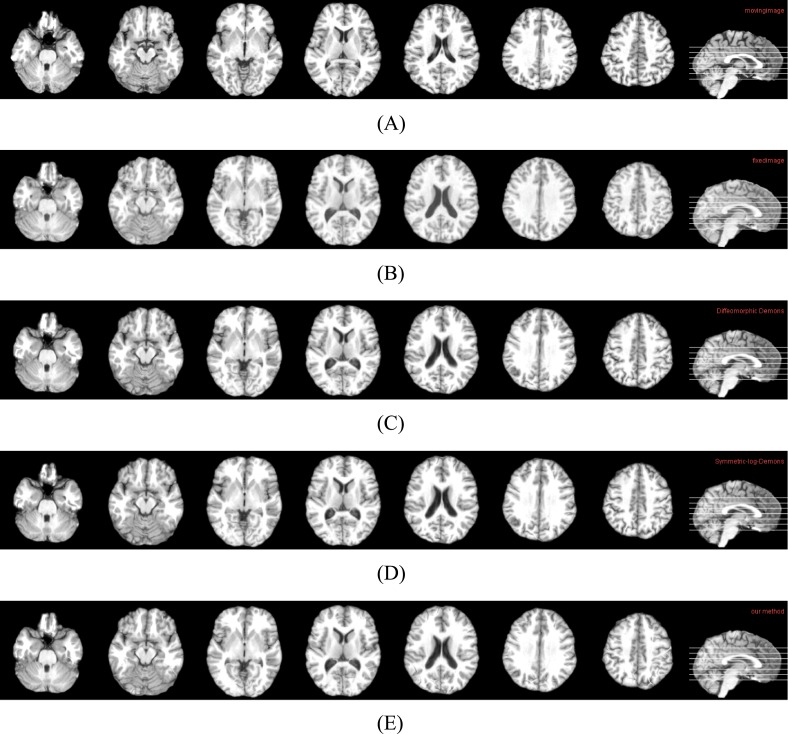
Cross-sectional views of data and registration results by three methods. (A) Cross-sectional views of moving image. (B) Cross-sectional views of fixed image. (C) Cross-sectional views of the result of Diffeomorphic Demons. (D) Cross-sectional views of the result of Symmetric-log-Demons. (E) Cross-sectional views of the result of our method.

**Fig 8:**
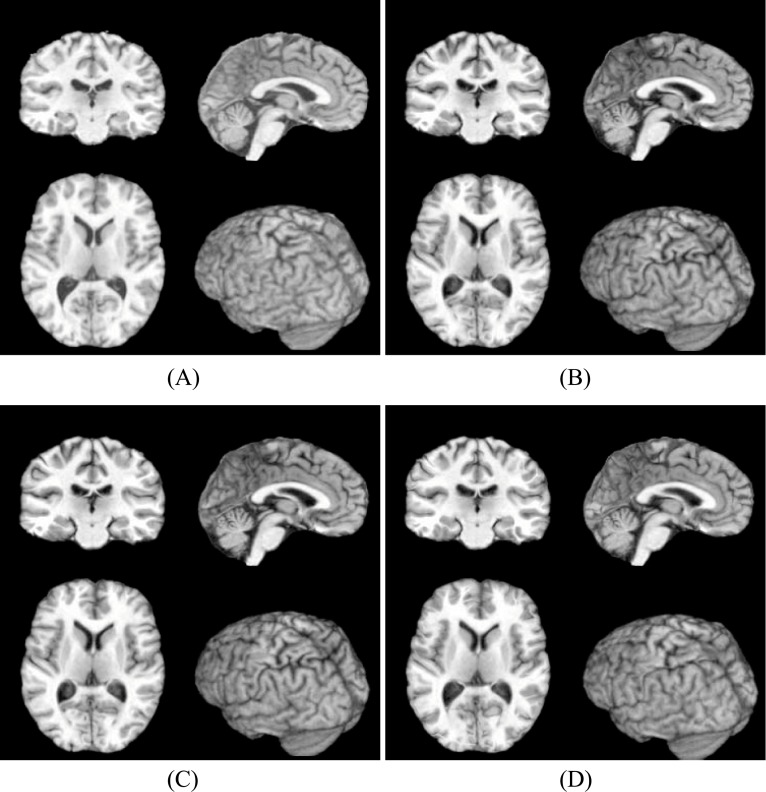
Fixed image and the registration results by three methods. (A) Fixed image. (B) Registration result by Diffeomorphic Demons. (C) Registration result by Symmetric-log-Demons. (D) Registration result by our method.

**Fig 9:**
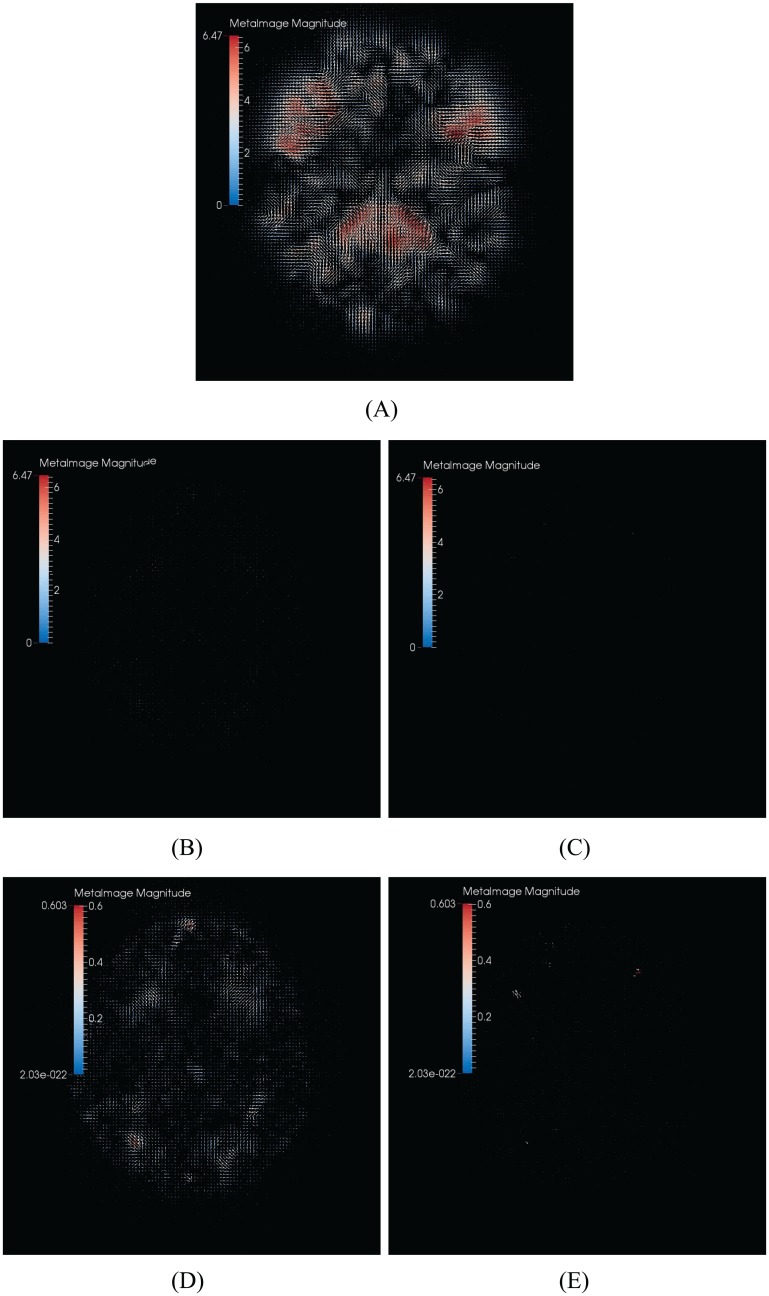
The combined deformations by three methods. (A) Diffeomorphic Demons. (B) Symmetric-log-Demons. (C) Our method. (D) Symmetric-log-Demons in 10 times magnification. (E) Our method in 10 times magnification.

By using two conventional nonlinear registration methods and our method, one group of results are shown in Figs 7, 8 and 9, and the quantitative results are shown in Table 2. Similar to the results on LONI data, our method still have some improvements at the aspect of precision as well as it preserves the deformations between two images Reciprocal. Concretely, in Fig 7, (A) and (B) are the cross-sectional images of moving image and fixed image, respectively. (C), (D) and (E) are the registration results from the moving image to fixed image by three algorithms. It is seen that all methods perform well. That is, (C), (D) and (E) are all similar to (B). It should be pointed out that there are still have some differences, which are shown in Fig 8. It is easy to find that our registration result (D) still has a slight improvement and is closer to the fixed image (A).

**Table 2:** Dice ratios of 83 ROIs by three methods.

	whole brain (average)
Diff. Demons	76.12 ± **3.3082**
Symmetric-Demons	76.04 ± 3.3797
Our method	**76.85** ± 3.4018

Furthermore, we show the combinations of the straight and backward deformation fields in Fig 9. From Fig 9(A), 9(B) and 9(C), the maximal magnitude is 6.47 mm. At this scale, (B) and (C) are almost black. That is, the combinations of the straight and backward deformation fields are closer to identities. Therefore, it is seen that both Symmetric-Demons and our proposed method are more accurate than the conventional Diffeomorphic Demons. But when we enlarge (b) and (c) with scale 10, the results are shown in Fig 9(D) and 9(E). From the Fig 9(D), the maximal magnitude of Symmetric-Demons is 0.603 mm, and the combined deformation field of our method is still close to an identity. That is, the deformation of our proposed method is closer to the diffeomorphism than the Symmetric-Demons, and therefore, our proposed method is more accurate.

**Fig 10:**
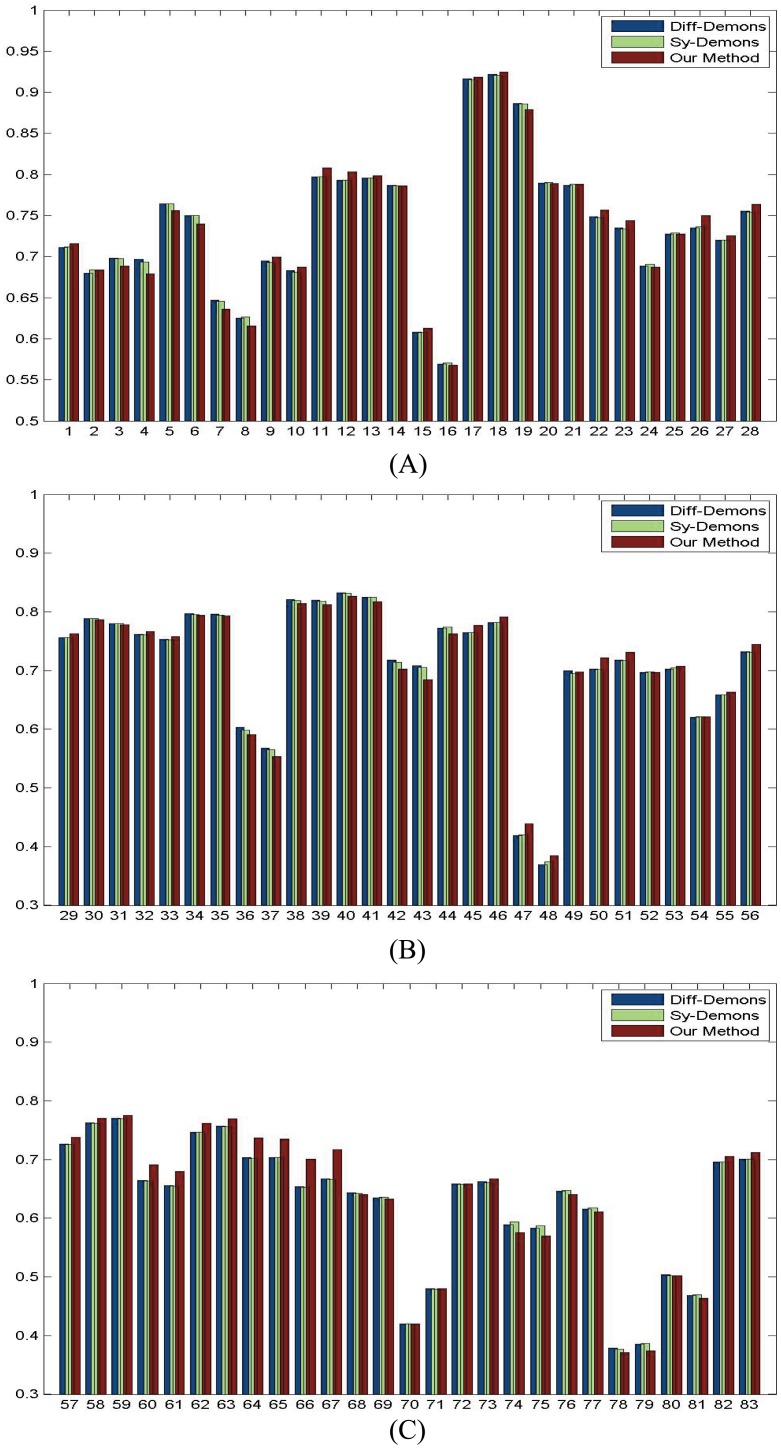
Dice ratios of 83 ROIs by three methods. (A) Dice ratios from 1st to 28th ROIs. (B) Dice ratios from 29th to 56th ROIs. (C) Dice ratios from 57th to 83th ROIs.

Finally, to quantitatively evaluate the registration accuracy, we calculate the Dice ratios of 83 ROIs by three methods shown in Fig 10, and the total average of whole brain is list in Table 2. It is seen that our proposed method has a slight improvement.

In general, from the numerical results, our proposed method not only improves the accuracy of the registration slightly, but also assures that the deformation is an actual diffeomorphism. In addition, by our method, we can get the deformation field and its inverse between two images at once.

## 5 Conclusion

In this paper, we considered the nonlinear image registration problem, especially, the invertibility of deformation field. First, we improve the registration model by using the bi-direction metric and introducing a regular constraint to assure that the deformation is a real diffeomorphism. Second, we decompose the model into two subproblems by the alternative iteration strategy. Then, inspired by the Diffeomorphic Demons algorithm, we established a novel registration algorithm, in which a new closed form for the velocity of deformation in each step is calculated as well as a multi-scale method is adopted. Finally, several numerical results on two real data validated that the propose method not only improved the accuracy of registration slightly, but also assured that the deformations between images are the actually invertible. In addition, by our method, we can gain the deformation field and its inverse between two images simultaneously.
